# How peer coaching can contribute to doctors’ development as clinical supervisors: an interview study

**DOI:** 10.1186/s12909-025-07660-3

**Published:** 2025-07-19

**Authors:** Erik Myrberg, Maria Henningsson, Catharina Tennerhed, Mats Wahlqvist, Matilda Liljedahl

**Affiliations:** 1 Institute of Clinical Sciences, Sahlgrenska Academy, University of Gothenburg, Gothenburg, Sweden; 2 Department of Rheumatology, Sahlgrenska University Hospital, Gothenburg, Sweden; 3 Department of Pediatrics, The Queen Silvia’s Children’s Hospital, Sahlgrenska University Hospital, Gothenburg, Sweden; 4 Department of Research, Education and Development, Sahlgrenska University Hospital, Gothenburg, Sweden; 5 School of Public Health and Community Medicine, Institute of Medicine, Sahlgrenska Academy, University of Gothenburg, Gothenburg, Sweden; 6 Department of Oncology, Sahlgrenska University Hospital, Gothenburg, Sweden

**Keywords:** Peer coaching, Faculty development, Peer feedback, Clinical supervision, Critical friends

## Abstract

**Purpose:**

Clinical supervisors work in demanding, complex environments, and faculty development activities should prepare them for these challenges. This study investigated peer coaching in the clinical setting as a faculty development activity for clinical supervisors. The study aimed to explore how peer coaching is enacted and how peer coaching may support clinical supervisors’ development.

**Methods:**

A realist evaluation study was conducted, involving qualitative interviews with 14 doctors who had participated in a peer coaching activity. Data were analyzed using a realist-informed reflexive thematic analysis.

**Results:**

Themes that describe the conditions, learning behaviors, and potential learning outcomes of peer coaching were developed. A necessary condition for effective peer coaching was “Practicing supervision under safe circumstances.” Peer coaching was characterized by “Exploring a teaching strategy,” “Handling authentic complexity,” “Reflecting during observation,” “Receiving credible feedback,” and “Contrasting educational situations.” Potential outcomes included an increased “Awareness of the student’s perspective” and that “Supervision becomes a shared concern.”

**Conclusion:**

The feedback component of peer coaching appears to support learning primarily by promoting reflective observation and initiating open, collegial discussions. Peer coaching can be conceptualized as reciprocal direct observation and discussions between equal colleagues, with mutual learning as the primary purpose. The contextually relevant influences on psychological safety are key factors to consider in implementing successful peer coaching.

**Clinical trial number:**

Not applicable.

**Supplementary Information:**

The online version contains supplementary material available at 10.1186/s12909-025-07660-3.

## Introduction

Clinical supervisors work in demanding environments and often need to balance the interests of patient care, research, and teaching. How clinical supervision in health professions education is enacted and affects medical students is complex, and the workplace’s influence is substantial [1]. A key challenge in transferring knowledge from faculty development programs is the competing demands of healthcare and education, along with the time constraints of the complex clinical environment [2]. In a systematic review of effective faculty development programs, several key features have been identified, such as opportunities for practical application, feedback, and reflection [3]. There is growing recognition of the need to investigate faculty development in the workplace and collaboration between educators to achieve high-quality education [4, 5].

Peer support models can be effective and feasible for training clinical supervisors [6], as they do not demand much senior faculty involvement. One such model is peer coaching, which has been implemented in varying forms but often constitutes a pre-observation meeting, direct observation of teaching, and subsequent feedback between colleagues [7]. Peer coaching participants have reported an increased awareness of their habits, adding that they appreciate receiving personalized teaching tips and being exposed to new teaching techniques [8]. A randomized trial on residents as teachers showed a higher degree of transfer of skills to the workplace among participants in a peer coaching program compared to participants in a traditional workshop [9].

In the field of higher education, faculty development approaches involving peer support usually include a pre-observation meeting, direct observation, reflection, and feedback [10]. Gosling used the term “reciprocal peer coaching” to describe activities where participating teachers exchange feedback and are, thus, both observers and acting teachers [11]. In the setting of the continued education of school teachers, Joyce and Showers have demonstrated considerably higher degrees of skills transfer from peer coaching interventions than historical controls from other forms of continued education [12].

In summary, the literature on peer coaching contributes some tentative insights regarding the value of practicing new skills in the clinical environment and feedback from a colleague. The literature has yet to fully explore how peer coaching can promote the development of clinical supervisors, including how contextual factors shape the implementation and effectiveness of peer coaching activities.

## Aim

The purpose of the study was to understand how peer coaching can be effectively implemented to support the transfer of learning from faculty development into clinical practice. Specifically, the study aimed to explore how peer coaching is enacted and how peer coaching may support clinical supervisors’ development.

## Materials and methods

### Research design

The present study was a qualitative interview study with doctors in specialist training participating in a peer coaching activity as part of a course in clinical supervision. A realist evaluation approach [13] was taken due to its potential strengths in developing a more nuanced and context-sensitive understanding of social phenomena [14]. Realist evaluation is an application of the critical realism paradigm [15]. Critical realists take a positivist ontological stance, meaning that data created through different methods can be combined to further the understanding of a single underlying reality. However, the constructivist epistemology of critical realism entails that theories are corrigible and fallible [16]. These paradigmatic tensions are handled by distinguishing the underlying reality from what can be observed and known, which are referred to as different ontological levels [15]. Observable events like student behavior or learning outcomes may be explained by so-called underlying mechanisms on another level, such as the psychological. Mechanisms may not be observable but can be inferred through data analysis in the light of the context and theory. This study utilized an abductive strategy where theory is tested against new data. Thus, previous peer coaching studies informed data generation and interpretation but were assumed to explain only certain aspects of the phenomenon [16].

### Context

**Fig. 1:**
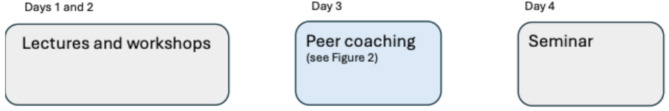
Overview of the course, which included peer coaching

The study’s setting was an introductory course in clinical supervision, illustrated in Fig. 1, which included a peer coaching activity. The introductory course is mandatory for doctors undergoing specialist training in the region and consists of four full days of studies [17].

**Fig. 2:**
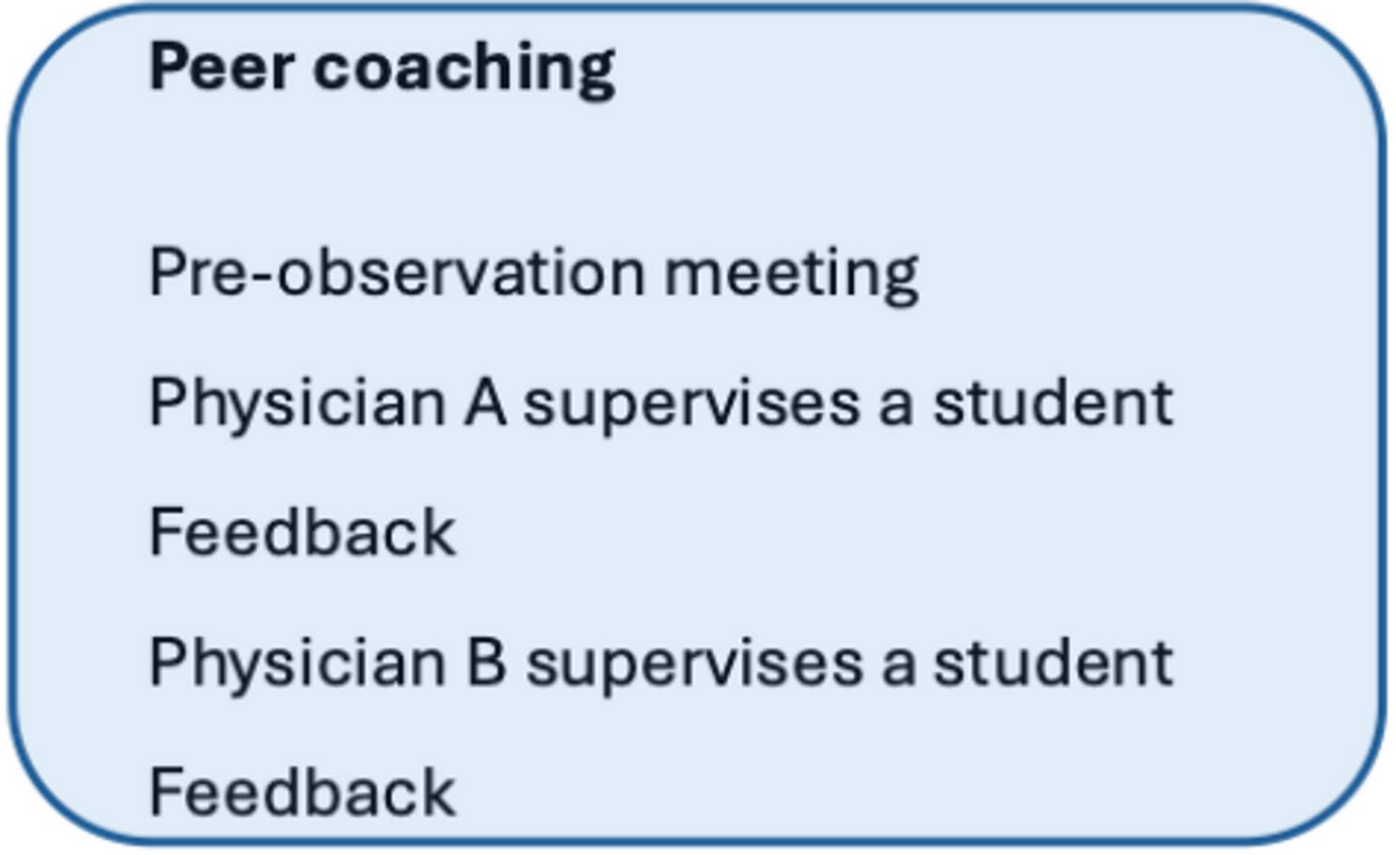
The peer coaching activity

The first two days of the introductory course include lectures and workshops on the medical curriculum, constructive alignment, clinical supervision, feedback, and small group learning. The third day comprises the peer coaching activity (Fig. 2) and a short written reflective report. The report is mandatory, but the course participants’ performance as supervisors is not assessed by course leaders. The final day includes a seminar about the course participants’ conclusions from the peer coaching activity and a workshop on teaching development.

The peer coaching activity in the course was designed based on the *collegial consultation with critical friends* model [18]. This peer coaching model can be seen as an application of experiential learning [19] where collegial feedback on supervision in the workplace is intended to enhance the participants’ reflection and planning of future supervisor strategies. Course participants are divided into dyads and instructed as follows (Fig. 2): Each dyad holds a pre-observation meeting to formulate specific observational questions on which they wish their colleague base their feedback. They then visit each other’s workplaces—one participant supervises a medical student or junior doctor, while the other focuses on providing feedback based on the observation questions. Participants take turns, each supervising and observing once.

### Data generation

The team comprised five researchers with experience in facilitating peer coaching activities. They had diverse backgrounds—health professions education researchers, faculty developers, medical doctors, clinical supervisors, and one was a schoolteacher. An interview guide was created for the purpose of the present study, see supplemental file #1. The interview guide comprised mainly broad and open-ended questions, allowing participants to uniquely describe their experiences [20]. The interview guide included initial questions about the sequence of events and interactions. The interviewer could then choose to explore these events further regarding how they affected learning [20]. The collective experience of the research team was used to formulate questions on areas such as the phases of experiential learning [19] and the peer relationship. These initial ideas were assumed to be relevant yet insufficient to explain the outcomes of peer coaching, congruent with an abductive realist evaluation strategy [13].

Course participants were invited to participate in the study after finishing the course and receiving course diplomas to mitigate social desirability bias [21]. Those who showed interest received information about the study, gave their written informed consent, and were included in the study. Interviews were performed by EM or MH 2–6 weeks after the peer coaching activity. EM had a previous personal relationship with two research participants, so they were interviewed by MH instead. Research participants could presumably be those who were particularly interested in clinical supervision, and the interviewers had experience facilitating peer coaching. Therefore, an interactionistic interview approach was chosen where the interview is considered a collaborative discussion during which an understanding is co-created [22].

The team deemed the quality of the interview dialogue as high, and data were assessed to have sufficient information power [23] to answer the research question after 14 interviews. During data collection, the interview guide was reviewed continually. No major revisions were made, but as the value of observing a peer became apparent in the first few interviews, the observation was subsequently explored more thoroughly.

The research participants were 29–38 years old and all had some prior experience as clinical supervisors. Twelve were undergoing specialist training, and two were specialists. Nine worked at a university hospital, two in regional hospitals, and three in primary care. Nine medical specialties were represented. Eleven were female, and three were male, compared to a slight majority of women among Swedish doctors in specialist training.

Twelve interviews were performed in person and two via video call due to geographical distance. Interviews were 47–65 min long, and they were audio-recorded and transcribed verbatim by the respective interviewer. Data were pseudonymized during transcription.

### Data analysis

Data were analyzed through reflexive thematic analysis [24] in an iterative process of using the researchers’ collective experience and literature to reflect on the meaning of data. EM extracted meaning units, including what was described as learning opportunities and learning outcomes by participants or interpreted as such by EM. Data extraction from the first four interviews was cross-checked with the team to avoid excluding any specific perspective. Some meaning units were merely condensed, and some were abstracted using educational terms. Abstraction was performed only when the abstracted meaning units remained close to the data and aligned with common definitions of the terms used to promote interpretative validity [25, 26].

Realist evaluation stipulates that educational interventions produce their outcomes through interaction with the context in a way that affects the reasoning and actions of the participants [13]. This informed the development of three research questions and the development of themes on these three levels (later shown in Table 1). Themes on the level of participants’ actions were developed to answer the question, “*What constitutes peer coaching?”* Themes on the level of learning outcomes were created to answer the question, “*What can be learned from peer coaching?”* The team analyzed these themes and the entire dataset to find possible explanations and mechanisms behind the other themes [26]. Thus, one theme was created to answer the question, “*What is necessary for effective peer coaching*?”

The first author kept a reflective journal and noticed how his practical and emotional involvement in faculty development might have steered his attention toward more complex learning outcomes during interpretation. Several other factors may have contributed to the same direction, such as the data collection time after the course completion, voluntary research participants who were potentially particularly engaged, and the possibility that the interview process may have contributed to participants’ reflections. The research team discussed whether these factors might obscure results around more simple and practical teaching skills that junior supervisors sometimes seek [27]. However, the participants’ detailed descriptions of how specific events [20] had led them to certain conclusions suggested that the learning outcome themes were indeed potential results of peer coaching. Data were reexamined, but the group could not create any more well-supported themes.

## Results

The presentation of the findings below follows the hypothesized causal sequences identified in the analysis: conditions, actions and learning outcomes. The question “What is necessary for effective peer coaching?” was answered by the theme “*Practicing supervision under safe circumstances*”. Five themes were created that answered the question, “What constitutes peer coaching?”: “*Exploring a teaching strategy*,” “*Handling authentic complexity*,” “*Reflecting during observation*,” “*Receiving credible feedback*,” and “*Contrasting educational situations*.” Finally, two themes answering the question “What can be learned from peer coaching?” were created: “*Awareness of the student’s perspective*” and “*Supervision becomes a shared concern*.” (Table 1).

**Table 1:** Overview of the research questions and themes

Research questions	What is necessary for effective peer coaching?	Whtat constitutes peer coaching?	What can be learned from peer coaching?
**Themes**	Practicing supervision under safe circumstances	Exploring a teaching strategy	Supervision becomes a shared concern
		Handling authentic complexity	Awareness of the student's perspective
		Reflecting during observation	
		Receiving credible feedback	
		Contrasting educational situations	

### Practicing supervision under safe circumstances

Participants described how the peer coaching situation had a low perceived risk of negative outcomes compared to previous experiences of being observed by a superior. ***Participant #10***: ***“If it is someone more experienced than me who will—I do not want to say ‘assess’ me***,*** but who is supervising me—then there is a different type of pressure to perform.”*** Most described peer coaching as an activity where they worried little about their own performance and how this was uncommon and appreciated. ***#2: “A much more relaxed situation***,*** where you can sort of think independently as well.”*** Clinical supervision was described as important and stimulating but potentially demanding. During this challenge, the presence of a peer on a similar level could be perceived as something positive—as friendly support.

The experience of safety was especially facilitated by personal rapport, mutual planning, and the absence of judgment. The analysis indicated that the perception of safe circumstances was an underlying factor in all other themes, as it influenced participants’ respective interactions.

### Exploring a teaching strategy

Participants described how they took an experimental attitude because they were less concerned with their own performance than they were used to. Therefore, they dared to explore new supervision tasks or styles inspired by their respective peer or by the previous supervision course content. This was expressed with words like “attempting” and “testing” and by some participants further emphasizing this with a light tone of voice. ***#9: “It did not work out as planned***,*** but that was okay because we both learned something.”*** The analysis indicated that this experimental attitude was a result of trust within the dyad of peers. ***#5: “We were there for each other.”***

### Handling authentic complexity

Overall, participants described their supervision encounters as authentic and emphasized this as a key factor in the perceived value of the peer coaching activity. The participants acknowledged the need to consider medical, pedagogical, and contextual factors, such as the lack of dedicated teaching spaces, during interactions with students. Some had planned to use specific feedback models but described how they had to “adapt” or “adjust” their approaches to navigate the unpredictable clinical environment and respond to students as individuals. This was perceived as immediately relevant to future teaching tasks. ***#8: “It becomes a challenge when you are put in a situation you have not controlled***,*** then the learning curve becomes completely different”.***

Some mentioned how they tried to make the activity meaningful by seeking supervision situations with common clinical situations. Others did not intentionally choose any especially suitable situation. ***#2: “We were pretty open to sort of taking on whoever would come.”.*** The analysis indicated that feeling safe promoted authenticity, as participants primarily chose or created supervision situations described as similar to upcoming supervisor tasks. Supervision encounters recognized as authentic also seemed to increase the perceived value of observation and feedback.

### Reflecting during observation

Participants described observing their peers as an opportunity to reflect on the actions and feelings of both the peer supervisor and the student. They reported that they took careful notes on specific phrases and body language to tailor their feedback effectively. ***#12: “I could lean back and observe the interaction and interplay and what my colleague did. And sure***,*** we did it a bit differently. But he did not do anything wrong***,*** only differently than what I would have done… When I sat and assessed him***,*** it was more like I thought***,*** ‘Well***,*** I guess you can do it like that as well!’ You elevate yourself a bit***,*** gain some perspective.”*** Participants described that they could focus on the supervision when they, as observers, did not have to take medical responsibility for the patient. Some participants could not recall specific details about their own supervisory experiences but could provide detailed descriptions of their peers’ interaction with their students. As such, the appreciation for observing was particularly apparent, and all participants expressed that observing was more valuable than teaching under observation.

### Receiving credible feedback

Participants universally reported that the feedback they received had felt non-threatening, and most found it valuable. An especially important reason for this described by participants was the perception that the peer had been an attentive observer. Another contributing factor was the sharing of goals during a pre-observation meeting, where mutual values regarding patient care and supervision were discussed. ***#13: “I trust his feedback because I know that he cares about his job and about his students. And he cares about me.”*** Some participants could not report what specific feedback they had received and described nervousness and a complex situation in the clinical environment as causes. However, they were able to report on their conclusions and plans for future strategies.

### Contrasting educational situations

Participants described their general peer coaching experience as a collegial discussion around their varying students, teaching styles, learning environments, and how strategies could be adapted for different situations. ***#8: “It worked so differently when we tried to make the interns reflect!… The second one did not seem to have received any supervision… We finally asked him about his previous experiences***,*** and he had not experienced it except for sort of an interrogation situation. And then we understood that it was new to him and maybe a skill that this intern needed to practice.”*** Participants articulated similarities and differences which they had observed and used words like “meta” and *“*perspective” to describe their own learning process. ***#10: “But after seeing different students and patients***,*** and every student got different advice—indirectly***,*** that is tailored feedback. I may not have seen it that way if it were only me***,*** if I had not seen the others.”*** These descriptions indicated that comparing different situations helped participants draw more general conclusions around learning and supervision.

### Increased awareness of the student’s perspective

One theme in the learning outcomes reported by participants was an increased focus on the student’s situation. Some participants mentioned having received helpful feedback on how the students reacted to their supervision style and comparing students’ roles at different clinics. Even more apparent in the data was the value of observation. ***#3: “I reflected on how exposed you must be as a student.”*** Participants articulated the complexity of the educational context and described envisioning themselves in the student’s role. Furthermore, they described observing as a rare opportunity to analyze the encounter from the learner’s perspective. According to participants authentic situations where students were engaged in their own curricular goals were particularly compelling to observe. For example, observers had noted the student’s reaction to assessments, which added a sense of importance to the encounter and resonated emotionally with participants. Some discussed their own experience as a learner with their peer and drew parallels between themselves and the students since they themselves were also learners in the peer coaching activity. ***#8: “He supervised me the same way. I got to assess what I had done well***,*** what did not go so well and what I could improve***,*** what goals I had myself as a supervisor. And I found that really helpful.”***

### Supervision becomes a shared concern

Participants reported how the reciprocal feedback started a free discussion around the subject of clinical supervision and compared it to a discussion about medical cases. ***#2: “It felt like a sort of collaboration around it… For example—Here is a problem around clinical supervision. I find this difficult; how can one solve this***?***”*** Participants frequently used the pronoun “we” when referring to their experiences and conclusions. ***#6: “Each of us taught only once***,*** but the second time***,*** we developed even further.”*** They specified their particular challenges as supervisors and how they had appreciated discussing them with a peer. ***#8: “We are both in specialist training and have similar experiences—know how it is on this middle level when you’re not an attending and not completely new. You have to take responsibility but can’t really make the decisions. It is hard!”*** Furthermore, they described how sharing the challenge of clinical supervision could have an emotional impact. ***#5: “It’s validating in a way. Regardless of different specialties***,*** different phases and that we are at different stages of our training***,*** that lots of these challenges are the same… Then you don’t feel as alone in these issues.”*** It was reported how seeing students in different clinics helped participants see the value of collaboration between supervisors. In conclusion, the analysis indicated that the supportive relationship, comparisons and discussions during peer coaching could potentially help participants turn an individual difficulty into a shared responsibility.

## Discussion

To address the challenge of transfer from faculty development activities, this study aimed to investigate how peer coaching can contribute to doctors’ development as clinical supervisors. Results suggest that peer coaching can promote collaboration between supervisors and raise awareness of the student’s perspective. The authenticity of situating peer coaching in the clinical environment has been described as one of its potential strengths [6]. Our study proposes that *practicing supervision under safe circumstances* is a key feature of successful peer coaching, and our results indicate a connection between the feeling of safety and perceived authenticity, as safe participants dared to seek authentic supervision situations, which they particularly valued. The results seem to emphasize the value of contrasting different educational situations to draw more general conclusions, and from a social-cognitive perspective, such comparisons are a key step in developing transferrable knowledge [28]. Further, our results detail how successful peer coaching can inspire exploratory and collaborative behaviors between participants during the activity.

The explorative behavior described by our participants can be understood as risk-taking facilitated by psychological safety. Psychological safety describes conditions where members of a work team feel safe to take risks, bring up tough issues, and seek help [29]. Such behaviors can significantly support learning between professionals [29] and in health professions education [30]. Tsuei et al. elaborated on how psychological safety can manifest as *educational safety*, thus allowing a *wholehearted focus on learning* [31]. Beyond grading, other potential consequences of performance may influence participants’ focus during peer coaching. An example from higher education is that participants may perceive peer coaching as a peer review of teaching that affects promotion decisions [11], which may hinder learning. Additionally, an American peer coaching program in surgical skills identified perceived economic competition and fear of legislative action as obstacles to learning [32]. By contrast, results in this study suggest that participants experienced peer coaching as a non-threatening situation, which stimulates open and free discussions.

Learners who are being observed and receive feedback may experience the situation as an assessment. It has been increasingly acknowledged how learners undergoing formative assessments may perceive the stakes as high, thus threatening psychological safety even in the absence of summative components. The terms high-stakes and low-stakes assessments widen the perspective and encompass how learners are affected not only by formal grading but also by the emotional consequences of assessments. Such emotional aspects are impression management [33] and self-esteem based on social comparisons [31]. Our results showed that peer coaching participants experienced the activity as non-evaluative, which allowed them to focus on learning rather than on performance. Based on this study, we therefore suggest that peer coaching implemented with high psychological safety can be regarded as a low stake assessment. Hence, if the primary goal of peer coaching is the development of clinical supervisory competence, educators should implement peer coaching with high psychological safety.

Although there is no unified definition of peer coaching in health professions education, previous literature often describes it in terms of the instruction that participants receive from faculty developers, such as direct feedback [7]. However, our results suggest that the conversations that may occur during peer coaching differ from historically common interpretations of feedback as information delivery from teacher to learner [34, 35]. Considering the value of observation and subsequent discussions, one interpretation is that the information delivery aspect of feedback may not be critical to peer coaching. Notably, well-cited school developers Joyce and Showers have emphasized reflection and collaborative experimentation rather than feedback in later iterations of their peer coaching programs [36].

From a realist evaluation perspective, understanding participants’ reasoning and actions is central to arguments about transferability. We suggest that faculty developers implement peer coaching in ways that are perceived as safe by participants. Furthermore, the same types of peer interactions described in the present study – such as exploration and collegial discussions – should be promoted. In the faculty development course studied, summative assessment was conducted separately from the peer coaching component — a feature we believe supported both safety and exploration. It is likely that the promotors and barriers to psychological safety differ, at least in part, across contexts. Further inquiry could consider how peer coaching can be adapted to diverse settings and sustainably embedded within institutional education strategies.

In future studies, we recommend conceptualizing peer coaching for clinical supervisors as a reciprocal process involving direct observation and discussions between equal colleagues, with mutual learning as the primary purpose.

## Methodological considerations

We included a limited number of participants, likely resulting in inadequate representation of those indifferent to peer coaching. Moreover, our study does not explicitly address the potential challenges of peer coaching or its impact on practice. Additionally, we have limited data on the influence of contextual factors, and the Swedish healthcare and specialist training systems are relatively egalitarian from a global perspective. Swedish doctors in specialist training enjoy secure employment, and all our participants worked in tax-funded clinics. Peer coaching may operate differently in contexts where competitive or unequal relationships are more pronounced. Furthermore, the fact that teaching is not our participants’ primary professional task may influence their perceived need to perform under observation.

The research team’s experience developing peer coaching activities is a strength of the study. It enabled the interpretation of participants’ descriptions of specific actions as learning opportunities, which may enhance the transferability of results beyond individual experiences. Additionally, using an abductive realist evaluation approach allowed the researchers to incorporate existing theories while using participants’ voices to add detail and nuance.

## Conclusion

Based on this study, we suggest that faculty developers who wish to implement peer coaching aim to promote psychological safety. This can allow participants to focus on learning and may encourage explorative behaviors and collegial discussions, contributing to their development as clinical supervisors.

## Electronic supplementary material

Below is the link to the electronic supplementary material.

Supplementary Material 1

## Data Availability

Pseudonymized interview data are available from the corresponding author on reasonable request.

## References

[1] Liljedahl M, et al. Navigating without a map: how medical students interact with clinical learning environments. Stud High Educ. 2019;44(2):275–86.

[2] Elmberger A, et al. Contradictions in clinical teachers’ engagement in educational development: an activity theory analysis. Adv Health Sci Educ. 2019;24(1):125–40. 10.1007/s10459-018-9853-y PMC6373255 30284068

[3] Steinert Y, et al. A systematic review of faculty development initiatives designed to enhance teaching effectiveness: A 10-year update: BEME guide 40. Med Teach. 2016;38(8):769–86. 27420193 10.1080/0142159X.2016.1181851

[4] O’Sullivan PS, Irby DM. Reframing research on faculty development. Acad Med. 2011;86(4):421–8. 21346505 10.1097/ACM.0b013e31820dc058

[5] Steinert Y. Faculty development: from rubies to oak. Med Teach. 2020;42(4):429–35. 31769320 10.1080/0142159X.2019.1688769

[6] Campbell N, et al. Peer-supported faculty development and workplace teaching: an integrative review. Med Educ. 2019;53(10):978–88. 31238387 10.1111/medu.13896 PMC6771963

[7] Schwellnus H, Carnahan H. Peer-coaching with health care professionals: what is the current status of the literature and what are the key components necessary in peer-coaching? A scoping review. Med Teach. 2014;36(1):38–46. 24094039 10.3109/0142159X.2013.836269

[8] Carlson K, et al. Peer coaching as a faculty development tool: A mixed methods evaluation. J Grad Med Educ. 2020;12(2):168–75. 32322350 10.4300/JGME-D-19-00250.1 PMC7161339

[9] Parrott S, Dobbie A, Chumley H. Peer coaching shows promise for residents as teachers. Fam Med. 2006;38(4):234–5. 16586162

[10] Esterhazy R, et al. Moving beyond peer review of teaching: A conceptual framework for collegial faculty development. Rev Educ Res. 2021;91(2):237–71.

[11] Gosling D. Collaborative peer-supported review of teaching. Dordrecht: Springer Netherlands; 2014. pp. 13–31.

[12] Joyce B, Showers B. *Designing training and peer coaching: Our needs for learning.* Student achievement through staff development, 2002: pp. 69–94.

[13] Pawson R. *Realistic evaluation*, ed. N. Tilley. 1997, London: SAGE.

[14] Wong G, et al. Realist methods in medical education research: what are they and what can they contribute? Med Educ. 2012;46(1):89–96. 22150200 10.1111/j.1365-2923.2011.04045.x

[15] Bhaskar R. A realist theory of science. Hassocks: Harvester P; 1978.

[16] Danermark B, Ekström M, Karlsson JC. Explaining society: critical realism in the social sciences. Routledge; 2019.

[17] Liljedahl M et al. Kollegahandledning – en effektiv modell Inom Handledarutbildning. Läkartidningen, 2021;118:2103. 34697791

[18] Lauvås P, Hofgaard Lycke K, Handal G. Kollegahandledning med Kritiska Vänner. Studentlitteratur; 2016.

[19] Kolb DA. Experiential learning: experience as the source of learning and development. 2 ed. Upper Saddle River, NJ: Pearson; 2015.

[20] Brönnimann A. How to phrase critical realist interview questions in applied social science research. J Crit Realism. 2022;21(1):1–24.

[21] Rubin HJ, Rubin IS. Qualitative interviewing: the Art of hearing data. Sage; 2011.

[22] Silverman D. *Interpreting qualitative data: Methods for analyzing talk, text and interaction*. 2006.

[23] Malterud K, Siersma VD, Guassora AD. Sample size in qualitative interview studies: guided by information power. Qual Health Res. 2016;26(13):1753–60. 26613970 10.1177/1049732315617444

[24] Braun V, Clarke V. Reflecting on reflexive thematic analysis. Qualitative Res Sport Exerc Health. 2019;11(4):589–97.

[25] Fletcher AJ. Applying critical realism in qualitative research: methodology Meets method. Int J Soc Res Methodol. 2017;20(2):181–94.

[26] Wiltshire G, Ronkainen N. A realist approach to thematic analysis: making sense of qualitative data through experiential, Inferential and dispositional themes. J Crit Realism. 2021;20(2):1–22.

[27] McLeod P, et al. Which pedagogical principles should clinical teachers know? Teachers and education experts disagree. Disagreement on important pedagogical principles. Med Teach. 2009;31(4):e117–124. 19404883 10.1080/01421590802335900

[28] Cheung JJH, Kulasegaram KM. Beyond the tensions within transfer theories: implications for adaptive expertise in the health professions. Advances in Health Sciences Education; 2022. 10.1007/s10459-022-10174-y 36369374

[29] Edmondson A. Psychological safety and learning behavior in work teams. Adm Sci Q. 1999;44(2):350–83.

[30] McClintock AH, et al. Psychological safety in medical education: A scoping review and synthesis of the literature. Med Teach. 2023;45(11):1290–9. 37266963 10.1080/0142159X.2023.2216863

[31] Tsuei SH-T, et al. Exploring the construct of psychological safety in medical education. Acad Med. 2019;94(11S Association of American Medical Colleges Learn Serve Lead: Proceedings of the 58th Annual Research in Medical Education Sessions):S28–35. 31365407 10.1097/ACM.0000000000002897

[32] Greenberg CC, et al. A statewide surgical coaching program provides opportunity for continuous professional development. Ann Surg. 2018;267(5):868–73. 28650360 10.1097/SLA.0000000000002341

[33] Schut S, et al. Stakes in the eye of the beholder: an international study of learners’ perceptions within programmatic assessment. Med Educ. 2018;52(6):654–63. 29572920 10.1111/medu.13532 PMC6001565

[34] Ramani S, et al. Feedback redefined: principles and practice. J Gen Intern Med. 2019;34(5):744–9. 30783881 10.1007/s11606-019-04874-2 PMC6502935

[35] Tavares W, et al. Learning conversations: an analysis of the theoretical roots and their manifestations of feedback and debriefing in medical education. Acad Med. 2020;95(7):1020–5. 31365391 10.1097/ACM.0000000000002932

[36] Joyce BR. In: Showers B, editor. Student achievement through staff development. 3 ed. Alexandria, Va.: Association for Supervision & Curriculum Development 2002.

