# Supplementary material for: How peer coaching can contribute to doctors’ development as clinical supervisors: an interview study
Source: BMC Med Educ. 2025 Jul 19;25:1085. doi: 10.1186/s12909-025-07660-3 (PMC12276701; doi:10.1186/s12909-025-07660-3)
Supplement: Supplementary file 1 — Supplementary Material 1 [file 12909_2025_7660_MOESM1_ESM.docx]

# Supplementary file 1

Interview guide

For how long have you supervised?

For how long have you worked as a physician?

Can you tell me about when you participated in peer coaching?

What happened?

- The planning
- Implementation
- The conversation afterward

Where did it take place?

Was it at your workplace?

With whom?

When you were observed by your colleague, how was that for you?

What was easy?

What was difficult?

When you received feedback from your colleague, how was that for you?

Can you describe the feedback you received?

How did you experience the feedback you received?

What do you think about the feedback?

How did it feel to receive that feedback?

When you observed your colleague and provided feedback, how was that for you?

What was easy?

What was difficult?

How was the feedback received?

Was there anything specific you learned from observing?

How was it for you to engage in peer supervision specifically with x?

How was your collaboration?

Now that you have experienced peer supervision—observing and supervising each other, how was it for you to experience peer supervision?

What did you feel you learned from this task?

Was there anything particularly educational about the peer supervision?

What did you take away from this experience?

Was there anything that surprised you?

Was there anything difficult about peer supervision?

Now that you have been part of peer supervision, what are your thoughts on it?

Is there anything else you would like to add?
